# Novel *ZEB2-BCL11B* Fusion Gene Identified by RNA-Sequencing in Acute Myeloid Leukemia with t(2;14)(q22;q32)

**DOI:** 10.1371/journal.pone.0132736

**Published:** 2015-07-17

**Authors:** Synne Torkildsen, Ludmila Gorunova, Klaus Beiske, Geir E. Tjønnfjord, Sverre Heim, Ioannis Panagopoulos

**Affiliations:** 1 Section for Cancer Cytogenetics, Institute for Cancer Genetics and Informatics, The Norwegian Radium Hospital, Oslo University Hospital, Oslo, Norway; 2 Centre for Cancer Biomedicine, Faculty of Medicine, University of Oslo, Oslo, Norway; 3 Department of Hematology, Oslo University Hospital, Oslo, Norway; 4 Department of Pathology, Oslo University Hospital, Oslo, Norway; 5 Institute of Clinical Medicine, Medical Faculty, University of Oslo, Oslo, Norway; Queen's University Belfast, UNITED KINGDOM

## Abstract

RNA-sequencing of a case of acute myeloid leukemia with the bone marrow karyotype 46,XY,t(2;14)(q22;q32)[5]/47,XY,idem,+?4,del(6)(q13q21)[cp6]/46,XY[4] showed that the t(2;14) generated a *ZEB2-BCL11B* chimera in which exon 2 of *ZEB2* (nucleotide 595 in the sequence with accession number NM_014795.3) was fused to exon 2 of *BCL11B* (nucleotide 554 in the sequence with accession number NM_022898.2). RT-PCR together with Sanger sequencing verified the presence of the above-mentioned fusion transcript. All functional domains of BCL11B are retained in the chimeric protein. Abnormal expression of *BCL11B* coding regions subjected to control by the *ZEB2* promoter seems to be the leukemogenic mechanism behind the translocation.

## Introduction

Acute myeloid leukemia (AML, also known as acute myelogenous leukemia or acute nonlymphocytic leukemia) is a malignancy of the myeloid line of blood cells, characterized by the rapid growth of abnormal immature white blood cells (blasts) that accumulate in the bone marrow and interfere with the production of normal blood cells. According to World Health Organization (WHO) criteria, a diagnosis of AML is established by demonstrating presence of 20% or more blasts in the blood and/or bone marrow [1]. AML is more common in adults, in whom the incidence rises steeply after the age of 55–60 years. The median age is 65 years and men are more often affected than women [2].

Chromosome banding analysis of bone marrow cells has revealed acquired clonal chromosomal rearrangements in most AML patients. The types of aberrations and the frequency with which they are found are influenced by factors such as age, previous treatment or other exposure to genotoxics, gender, ethnic or geographic origin, and constitutional genetics [3]. Structural chromosomal aberrations in AML such as translocations, inversions, and deletions often result in the generation of a fusion gene, i.e., a hybrid gene formed from two previously separate genes, which affects cellular pathways of myeloid maturation and proliferation [3]. In the WHO classification of AML (and myeloid neoplasms in general), the chromosomal rearrangements/fusion genes play an important role in the grouping of AMLs into diagnostic-prognostic-therapeutic entities; for example, rearrangements such as t(8;21)(q22;q22) (fusion gene *RUNX1-RUNX1T1*), inv(16)(p13.1q22) (fusion gene *CBFB-MYH11*), and t(15;17)(q22;q12) (fusion gene *PML-RARA*) define specific AML subsets and their finding is sufficient for an AML diagnosis regardless of the blast percentage in the blood or bone marrow [1]. The three above-mentioned chromosomal aberrations/fusion genes are also associated with favorable prognosis [3] whereas others, e.g., *KAT6A-CREBBP*, the result of a t(8;16)(p11;p13) chromosomal translocation, are associated with poor prognosis [4]. Furthermore, fusion genes can be the targets of specific molecular therapy, the paradigmatic example being the use of all-trans retinoic acid (ATRA)/arsenic trioxide (ATO) in the treatment of acute promyelocytic leukemia (APL) carrying the *PML—RARA* fusion which is key to APL leukemogenesis generated by t(15;17) [5]. Hence, the identification and characterization of novel leukemia-specific fusion genes and the study of their effects on cellular processes have clinical significance.

The “traditional” methodology to detect fusion genes in cancer begins with cytogenetic analysis to find the chromosomal rearrangement, followed by utilization of fluorescence in situ hybridization (FISH) techniques to find a probe which spans the chromosomal breakpoint. Eventually, molecular cloning is performed to localize the breakpoint more precisely and identify the genes fused by the chromosomal rearrangement. Although laborious, the above-mentioned sequential approach is quite robust and reliable and a number of fusion genes have been cloned by such means [3]. The introduction of next generation sequencing has opened up new possibilities to detect fusion genes in cancer [6]. The search for fusion genes presently in the main proceeds along two main axes, either using brute molecular genetic force (i.e., high throughput sequencing) in the common cancers where cytogenetic guidance is sparse at best, or by combination of cytogenetics and sequencing so that the karyotypic information can be used to select among the numerous candidate fusion genes offered by the latter method to find only those few that map to chromosomal breakpoints [7]. The actual presence of the fusion genes thus detected is then confirmed by other molecular genetic (PCR and Sanger sequencing analyses) and molecular cytogenetic methods (FISH).

We and others have used combinations of cytogenetics and RNA-sequencing to detect the fusion genes associated with acquired cancer-specific chromosomal rearrangements [7]. These fusion genes were considered to be primary tumorigenic events because the chromosomal rearrangements generating them were often seen as the sole aberration by cytogenetic analysis.

Recently, a recurrent t(2;14)(q22;q32) chromosomal translocation was reported in immature early T-cell precursor leukemia (ETP-ALL) [8]. Using FISH, the translocation was shown to affect the *BCL11B* and *ZEB2* loci on chromosome bands 14q32 and 2q22, respectively, resulting in overexpression of *ZEB2*. However, neither *BCL11B-ZEB2* nor *ZEB2-BCL11B* (nor their respective fusion transcripts) was demonstrated in that study.

We here report an AML case carrying the same t(2;14)(q22;q32) chromosomal translocation in bone marrow cells as that reported in ETP-ALL. Using RNA-sequencing, the translocation was shown to result in generation of a *ZEB2-BCL11B* fusion gene.

## Materials and Methods

### Ethical approval

The study was approved by the Regional Committee for Medical and Health Research Ethics, South-East Norway (REK Sør) http://helseforskning.etikkom.no). Written informed consent was obtained from the patient prior to his death. The consent sought from the participant included consent for the publication of his clinical details. The ethics committee’s approval included a review of the consent procedure. All patient information has been anonymized and de-identified.

### Patient

**Fig 1:**
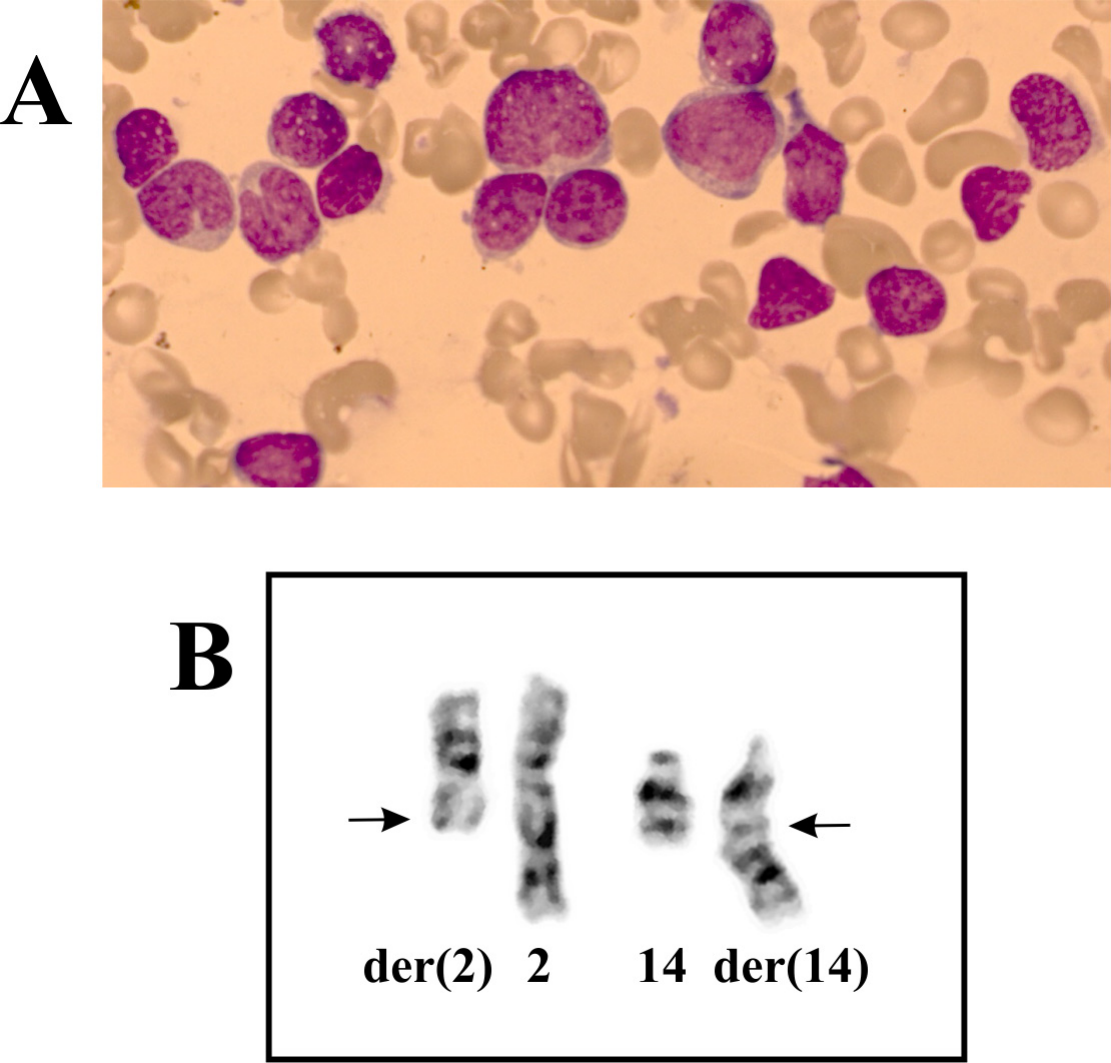
Hematological and cytogenetic data on the AML patient with t(2;14). A) Blasts without granulation and with relatively little cytoplasm in the patient´s bone marrow. B) Partial karyotype showing the der(2)t(2;14)(q22;q32) and der(14)t(2;14)(q22;q32) together with the corresponding normal chromosome homologs; breakpoint positions are indicated by arrows.

A 28-year-old man was transferred to our institution with a diagnosis of AML (Table 1 and Fig 1A). He presented with signs of infection (fever and cough) and a remarkable swelling on the neck with slight rubor and tenderness. Computer tomography (CT) demonstrated bilateral massive lymphadenopathy with necrosis. His AML turned out to be chemoresistant as he failed three induction courses (Table 1). Further leukemia-directed treatment was therefore discontinued, and the patient succumbed within short of infections.

**Table 1:** Hematological data and treatment on the AML patient with the t(2;14) chromosomal aberration.

Blood analysis		Smear		Molecular genetic analysis in BM		Immunophenotype in BM		Treatment
**Hb**	8.0 g/dL (13.4–17.0)	**BM**	90% blasts with a sparse cytoplasmic brim and no granulation	**Positive**	FLT3-ITD	**Positive**	Strongly CD34+ and CD117+ (88%)	1. Idarubicin 12mg/m^2^ day 1–3, cytarabin 200 mg/m^2^ day 1–7
**Plc**	291 x 10^9^/L (145–390)	**PB**	85% blasts	**Negative**	FLT3-TKD, EVI-1, CEBPalfa, NPM1, CBFB-MYH11, RUNX1-RUNX1T1	**Coexpression**	HLA-DR, CD38, CD13, CD71, CD7, Tdt	2. Cytarabin 1g/m^2^ x 2 day 1–6, amsakrin 120 mg/m^2^ day 4–6
**WBC**	29.7 x 10^9^/L (3.5–10)							3. Amsakrin 150 mg/m^2^ day 1–5, etoposide 110 mg/m^2^ day 1–5, cytarabin 200 mg/m^2^ day 1–5
**LD**	677 U/L (105–205)							

Hb, hemoglobin; Plc, platelet count; WBC, white bloood cells; LD, lactate dehydrogenase; BM, bone marrow; PB, peripheral blood.

### Control samples

Two controls were used: A previously reported AML with karyotype at diagnosis 46,XY,add(1)(p13),t(8;21)(p11;q22),der(16)t(1;16)(p13;p13)[9]/46,XY[1] which was shown to carry a cryptic *KAT6A-CREBBP* fusion gene [9], and human bone marrow total RNA (Clontech Laboratories, Inc., Mountainview, CA, USA). According to the company’s information, it is a mixture of normal bone marrow pooled from 6 male/female Caucasians, ages: 38–59.

### G-banding and karyotyping

Bone marrow cells were cytogenetically investigated by standard methods. Chromosome preparations were made from metaphase cells of a 24-hours culture, G-banded using Leishman stain, and karyotyped according to ISCN 2009 guidelines [10].

### RNA-sequencing

Total RNA was extracted from the patient’s bone marrow at the time of diagnosis using miRNeasy Mini Kit according to the manufacturer’s instructions (Qiagen Nordic, Oslo, Norway). Subsequently, total RNA was purified using QIAcube (Qiagen). The RNA quality was evaluated using the Experion Automated Electrophoresis System (Bio-Rad Laboratories, Oslo, Norway). The RNA Quality Indicator (RQI) was 7.8. Three μg of total RNA were sent for high-throughput paired-end RNA-sequencing at the Norwegian Sequencing Centre, Ullevål Hospital (http://www.sequencing.uio.no/). The RNA was sequenced using an Illumina HiSeq 2000 instrument and the Illumina software pipeline was used to process image data into raw sequencing data. The TruSeq Stranded mRNA sample preparation protocol was used (http://support.illumina.com/downloads/truseq_stranded_mrna_sample_preparation_guide_15031047.ilmn) giving reads of a length of 100 base pairs. A total of 70 million reads were obtained. The quality of the raw sequence data was assessed using FastQC software (http://www.bioinformatics.babraham.ac.uk/projects/fastqc/). Two softwares were used for the discovery of fusion transcripts: FusionCatcher (version 0.99.3a beta-April 15, 2014) with the associated ENSEMBL, UCSC, and RefSeq databases automatically downloaded by FusionCatcher (https://code.google.com/p/fusioncatcher/) [11] and ChimeraScan (https://code.google.com/p/chimerascan/) [12]. To verify further the fusion genes which were found by FusionCatcher and ChimeraScan, the “grep” command (http://en.wikipedia.org/wiki/Grep) was used to search the fastq files of the sequence data (http://en.wikipedia.org/wiki/FASTQ_format). Our “specific expression” was a sequence of 20 nucleotides at the fusion point, 10 bases upstream (5´-end gene), and 10 bases downstream from the junction (3´-end gene). The expressions were: "AGGAAAAACGCAGAGGCTGA" (type 1), "AGGAAAAACGAGGCTGACCA" (type 2), "GACTTCGCAGAGGCTGACCA" (type 3), and "AGGAAAAACGAGCACAAAAG" (type 4) for the four alternative fusion transcripts of *ZEB2-BCL11B* which were found with FusionCatcher (see S1 Table). The sequences obtained by “grep” were blasted against the human genomic plus transcript database (http://blast.ncbi.nlm.nih.gov/Blast.cgi) as well as the reference sequences NM_014795.3 (*ZEB2*) and NM_022898.2 (*BCL11B*).

#### RT-PCR analysis

The primers used for PCR amplification and Sanger sequencing are listed in Table 2.

**Table 2:** Primers used for PCR amplification and Sanger sequencing analyses.

Name	Sequence (5´->3´)	Direction	Position / Exon	Reference Sequence	Gene
ZEB2-383F1	CACACTTCGCGGCTTCTTCATGCTT	Forward	383–407 / 1	NM_014795.3	*ZEB2*
ZEB2-485F1	CGAGTCCATGCGAACTGCCATCTG	Forward	485–508 / 2	NM_014795.3	*ZEB2*
ZEB2-672R1	TGGTCCAGAGGGTTGGCAATACCG	Reverse	695–672 / 3	NM_014795.3	*ZEB2*
ZEB2-985R1	GCTGACTGCATGACCATCGCGTTC	Reverse	985–1008 / 5	NM_014795.3	*ZEB2*
BCL11B-464F1	CTCCGGGCGATGCCAGAATAGATG	Forward	464–487 / 1	NM_022898.2	*BCL11B*
BCL11B-654R1	CACTGGCCACAGGTGAGCAGGTCA	Reverse	677–654 / 2	NM_022898.2	*BCL11B*
BCL11B-969R1	GCAGGAACCACGCGCTGTTGAAG	Reverse	991–969 / 3	NM_022898.2	*BCL11B*
ABL1-91F1	CAGCGGCCAGTAGCATCTGACTTTG	Forward	280–304 / 2	NM_005157.5	*ABL1*
ABL1-404R1	CTCAGCAGATACTCAGCGGCATTGC	Reverse	617–593 / 3	NM_005157.5	*ABL1*

For RT-PCR, one μg of total RNA was reverse-transcribed in a 20 μL reaction volume using iScript Advanced cDNA Synthesis Kit for RT-qPCR according to the manufacturer’s instructions (Bio-Rad Laboratories, Oslo, Norway). The 25 μL PCR volume contained 12.5 μL Premix Ex Taq DNA Polymerase Hot Start Version (Takara Bio Europe/SAS, Saint-Germain-en-Laye, France), 1 μL of cDNA, and 0.4 μM of each of the forward and reverse primer. The primer sets ZEB2-383F1/BCL11B-654R1 and ZEB2-485F1/BCL11B-654R1 were used to detect possible *ZEB2-BCL11B* fusion transcripts. The primer sets BCL11B-464F1/ZEB2-985R1 and BCL11B-464F1/ZEB2-672R1 were used to detect possible *BCL11B-ZEB2* fusion transcripts. To detect the expression of the normal *ZEB2* gene, the primer set ZEB2-383F1/ZEB2-985R1 was used. To detect expression of the normal *BCL11B* gene, the primer set BCL11B-464F1/BCL11B-969R1 was used. The quality of the cDNA synthesis was examined by amplification of a cDNA fragment of the *ABL1* gene using the primers ABL1-91F1 and ABL1-404R1. The PCR amplifications were run on a C-1000 Thermal cycler (Bio-Rad Laboratories) with an initial denaturation at 94°C for 30 sec, followed by 35 cycles of 7 sec at 98°C, 2 min at 68°C, and a final extension for 5 min at 68°C. Three μL of the PCR products were stained with GelRed (Biotium, Hayward, CA, USA), analysed by electrophoresis through 1.0% agarose gel, and photographed. The remaining 22 μL PCR products were purified using the MinElute PCR purification kit (Qiagen Nordic, Oslo, Norway) and sequenced at GATC Biotech (Germany, http://www.gatc-biotech.com/en/home.html). The BLAST software (http://blast.ncbi.nlm.nih.gov/Blast.cgi) was used for computer analysis of sequence data.

## Results

### G-banding

G-banding analysis of bone marrow cells at diagnosis showed two cytogenetically related subclones. In the first subclone, five cells carried the chromosomal translocation t(2;14)(q22;q32) as the sole cytogenetic abnormality (Fig 1B). In the second subclone, six cells carried an extra chromosome, presumably #4, and an interstitial deletion in chromosome 6 together with the above-mentioned translocation. Thus, G-banding analysis of bone marrow cells at diagnosis indicated that t(2;14)(q22;q32) was the primary cytogenetic abnormality. The resulting karyotype was 46,XY,t(2;14)(q22;q32)[5]/47,XY,idem,+?4,del(6)(q13q21)[cp6]/46,XY[4].

### RNA-sequencing

Using FusionCatcher on the raw sequencing data obtained from the Genomics Core Facility, 103 potential fusion transcripts were found (S1 Table), among them the *ZEB2-BCL11B*. Using ChimeraScan with the same fastq file, 263 potential fusion transcripts were found (S2 Table), among them the *ZEB2-BCL11B*. Neither FusionCatcher nor ChimeraScan found the reciprocal *BCL11B-ZEB2* (S1 and S2 Tables).

Further analysis of the fusion transcripts identified with the two softwares showed that FusionCatcher identified four types (types 1–4) of *ZEB2-BCL11B* (S1 Table). Type 1 corresponds to the fusion of exon 2 of *ZEB2* with exon 2 of *BCL11B*. Type 2 corresponds to the same fusion but without the first three, CAG, nucleotides of exon 2 of *BCL11B*. Type 3 corresponds to the fusion of exon 1 of *ZEB2* with exon 2 of *BCL11B* without the first three bases, CAG, of exon 2 of *BCL11B*. Type 4 corresponds to the fusion of exon 2 of *ZEB2* with nt 719 within exon 2 of *BCL11B*. Only the type 1 *ZEB2-BCL11B* fusion transcript was found by ChimeraScan (S2 Table).

In order to verify the fusion obtained with the FusionCatcher and ChimeraScan softwares, we used the “grep” command utility to search for expressions composed of 10 nt of *ZEB2* and 10 nt of *BCL11B* upstream and downstream of the fusion point (Table 3). Using the expression "AGGAAAAACGCAGAGGCTGA", which is composed of 10 nt, "AGGAAAAACG", from *ZEB2* and 10 nt, "CAGAGGCTGA", from *BCL11B*, 18 sequences were retrieved. With the expression "AGGAAAAACGAGGCTGACCA", which is composed of 10 nt, "AGGAAAAACG", from *ZEB2* and 10 nt, "AGGCTGACCA", from *BCL11B*, 4 sequences were retrieved. The expression "GACTTCGCAGAGGCTGACCA", which is composed of 10 nt, "GACTTCGCAG", from *ZEB2* and 10 nt, "AGGCTGACCA", from *BCL11B*, retrieved 2 sequences. Finally, "AGGAAAAACGAGCACAAAAG", which is composed of the 10 nt "AGGAAAAACG" from *ZEB2* and the 10 nt "AGCACAAAAG" from *BCL11B*, retrieved 1 sequence (Table 3).

**Table 3:** Sequences retrieved with the «grep» command using the expressions "AGGAAAAACGCAGAGGCTGA", "AGGAAAAACGAGGCTGACCA", "GACTTCGCAGAGGCTGACCA", and "AGGAAAAACGAGCACAAAAG" which correspond to type 1, type 2, type 3 and type 4 of *ZEB2-BCL11B* fusion transcript, respectively. The sequences of *BCL11B* are in bold.

Type (expression)	Sequences
Type 1 (AGGAAAAACG **CAGAGGCTGA**)	ATCCGCTCTTATCAATGAAGCAGCCGATCATGGCGGATGGCCCCCGGTGCAAGAGGCGCAAACAAGCCAATCCCAGGAGGAAAAACG**CAGAGGCTGACCAT**
	CAAACAAGCCAATCCCAGGAGGAAAAACG**CAGAGGCTGACCATGTGGAGGCCGCCATCCTCGAAGAAGACGAGGGTCTGGAGATAGAGGAGCCAAGTGGCC**
	CCAGGAGGAAAAACG**CAGAGGCTGACCATGTGGAGGCCGCCATCCTCGAAGAAGACGAGGGTCTGGAGATAGAGGAGCCAAGTGGCCTGGGGCTGATGGTG**
	ATCCCAGGAGGAAAAACG**CAGAGGCTGACCATGTGGAGGCCGCCATCCTCGAAGAAGACGAGGGTCTGGAGATAGAGGAGCCAAGTGGCCTGGGGCTGATG**
	CAGGAGGAAAAACG**CAGAGGCTGACCATGTGGAGGCCGCCATCCTCGAAGAAGACGAGGGTCTGGAGATAGAGGAGCCAAGTGGCCTGGGGCTGATGGTGG**
	AGGAGGAAAAACG**CAGAGGCTGACCATGTGGAGGCCGCCATCCTCGAAGAAGACGAGGAGCACAAAAGGAAGCAGTGTGGCGGCAGCTTGGGTGCCTGCTA**
	GGCGCAAACAAGCCAATCCCAGGAGGAAAAACG**CAGAGGCTGACCATGTGGAGGCCGCCATCCTCGAAGAAGACGAGGGTCTGGAGATAGAGGAGCCAAGT**
	GATCCGCTCTTATCAATGAAGCAGCCGATCATGGCGGATGGCCCCCGGTGCAAGAGGCGCAAACAAGCCAATCCCAGGAGGAAAAACG**CAGAGGCTGACCA**
	CGGTGCAAGAGGCGCAAACAAGCCAATCCCAGGAGGAAAAACG**CAGAGGCTGACCATGTGGAGGCCGCCATCCTCGAAGAAGACGAGGGTCTGGAGATAGA**
	ATGGCGGATGGCCCCCGGTGCAAGAGGCGCAAACAAGCCAATCCCAGGAGGAAAAACG**CAGAGGCTGACCATGTGGAGGCCGCCATCCTCGAAGAAGACGA**
	GGATGGCCCCCGGTGCAAGAGGCGCAAACAAGCCAATCCCAGGAGGAAAAACG**CAGAGGCTGACCATGTGGAGGCCGCCATCCTCGAAGAAGACGAGGGTC**
	GGCGGATGGCCCCCGGTGCAAGAGGCGCAAACAAGCCAATCCCAGGAGGAAAAACG**CAGAGGCTGACCATGTGGAGGCCGCCATCCTCGAAGAAGACGAGG** CAAGCCAATCCCAGGAGGAAAAACG**CAGAGGCTGACCATGTGGAGGCCGCCATCCTCGAAGAAGACGAGGGTCTGGAGATAGAGGAGCCAAGTGGCCTGGG**
	TATCAATGAAGCAGCCGATCATGGCGGATGGCCCCCGGTGCAAGAGGCGCAAACAAGCCAATCCCAGGAGGAAAAACG**CAGAGGCTGACCATGTGGAGGCC**
	AAGCAGCCGATCATGGCGGATGGCCCCCGGTGCAAGAGGCGCAAACAAGCCAATCCCAGGAGGAAAAACG**CAGAGGCTGACCATGTGGAGGCCGCCATCCT**
	ATGGCGGATGGCCCCCGGTGCAAGAGGCGCAAACAAGCCAATCCCAGGAGGAAAAACG**CAGAGGCTGACCATGTGGAGGCCGCCATCCTCGAAGAAGACGA**
	ATGGCGGATGGCCCCCGGTGCAAGAGGCGCAAACAAGCCAATCCCAGGAGGAAAAACG**CAGAGGCTGACCATGTGGAGGCCGCCATCCTCGAAGAAGACGA**
	CAAGCCAATCCCAGGAGGAAAAACG**CAGAGGCTGACCATGTGGAGGCCGCCATCCTCGAAGAAGACGAGGGTCTGGAGATAGAGGAGCCAAGTGGCCTGGG**
Type 2 (AGGAAAAACG **AGGCTGACCA**)	CAAGCCAATCCCAGGAGGAAAAACG**AGGCTGACCATGTGGAGGCCGCCATCCTCGAAGAAGACGAGGGTCTGGAGATAGAGGAGCCAAGTGGCCTGGGGCT**
	TCTGATCCGCTCTTATCAATGAAGCAGCCGATCATGGCGGATGGACCCCGGTGCAAGAGGCGCAAACAAGCCAATCCCAGGAGGAAAAACG**AGGCTGACCA**
	CAAGCCAATCCCAGGAGGAAAAACG**AGGCTGACCATGTGGAGGCCGCCATCCTCGAAGAAGACGAGGGTCTGGAGATAGAGGAGCCAAGTGGCCTGGGGCT**
	AAACAAGCCAATCCCAGGAGGAAAAACG**AGGCTGACCATGTGGAGGCCGCCATCCTCGAAGAAGACGAGGGTCTGGAGATAGAGGAGCCAAGTGGCCTGGG**
Type 3 (GACTTCGCAG **AGGCTGACCA**)	CTACAAACAAGACTTCGCAG**AGGCTGACCATGTGGAGGCCGCCATCCTCGAAGAAGACGAGGGTCTGGAGATAGAGGAGCCAAGTGGCCTGGGGCTGATGG**
	GCTTTTTCTTCTCACCATTTCTGGCCAAAACTACAAACAAGACTTCGCAG**AGGCTGACCATGTGGAGGCCGCCATCCTCGAAGAAGACGAGGGTCTGGAGA**
Type 4 (AGGAAAAACG **AGCACAAAAG**)	ATGAAGCAGCCGATCATGGCGGATGGCCCCCGGTGCAAGAGGCGCAAACAAGCCAATCCCAGGAGGAAAAACG**AGCACAAAAGGAAGCAGTGTGGCGGCAG**

Because the *ZEB2* and *BCL11B* genes map to chromosome bands 2q22 and 14q32, respectively, and the chromosomal translocation t(2;14)(q22;q32) was the primary cytogenetic aberration, we decided to investigate further the patient’s bone marrow for the presence of the *ZEB2-BCL11B* fusion transcript using molecular techniques. No other fusions were examined.

### Molecular Genetic Confirmation of the Fusions

**Fig 2:**
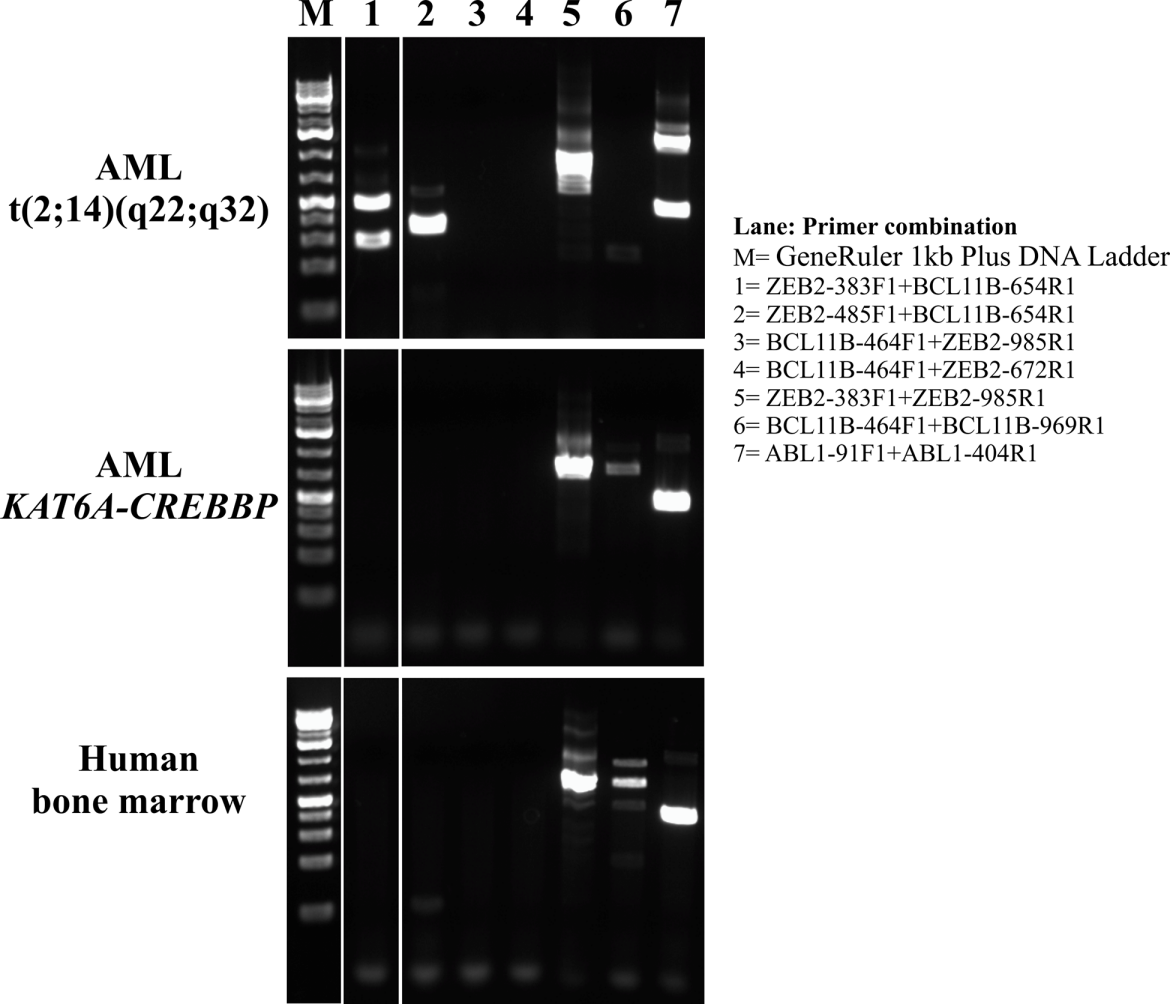
RT-PCR amplification. Gel electrophoresis for the *ZEB2-BCL11B*, *BCL11B-ZEB2*, *ZEB2*, *BCL11B*, and *ABL1* cDNA transcripts of AML patient with t(2;14), control AML patient with *KAT6A-CREBBP* fusion, and normal bone marrow.

PCR with the primers ABL1-91F1 and ABL1-404R1 amplified a 338 bp *ABL1* fragment from the cDNAs of the AML-control (patient with *KAT6A-CREBBP* fusion) and the normal bone marrow (Fig 2). From the cDNA of the patient with t(2;14), two fragments were amplified: 338 bp and 901 bp in size, respectively. The 338 bp fragment corresponds to the processed mRNA (exon 2/exon 3 of *ABL1*) while the 901 bp fragment corresponds to the presence of an unspliced intron 2 (exon 2-intron 2- exon 3) of *ABL1*. The amplified cDNA fragments of *ABL1* indicated that the synthesized cDNAs were of good quality.

**Fig 3:**
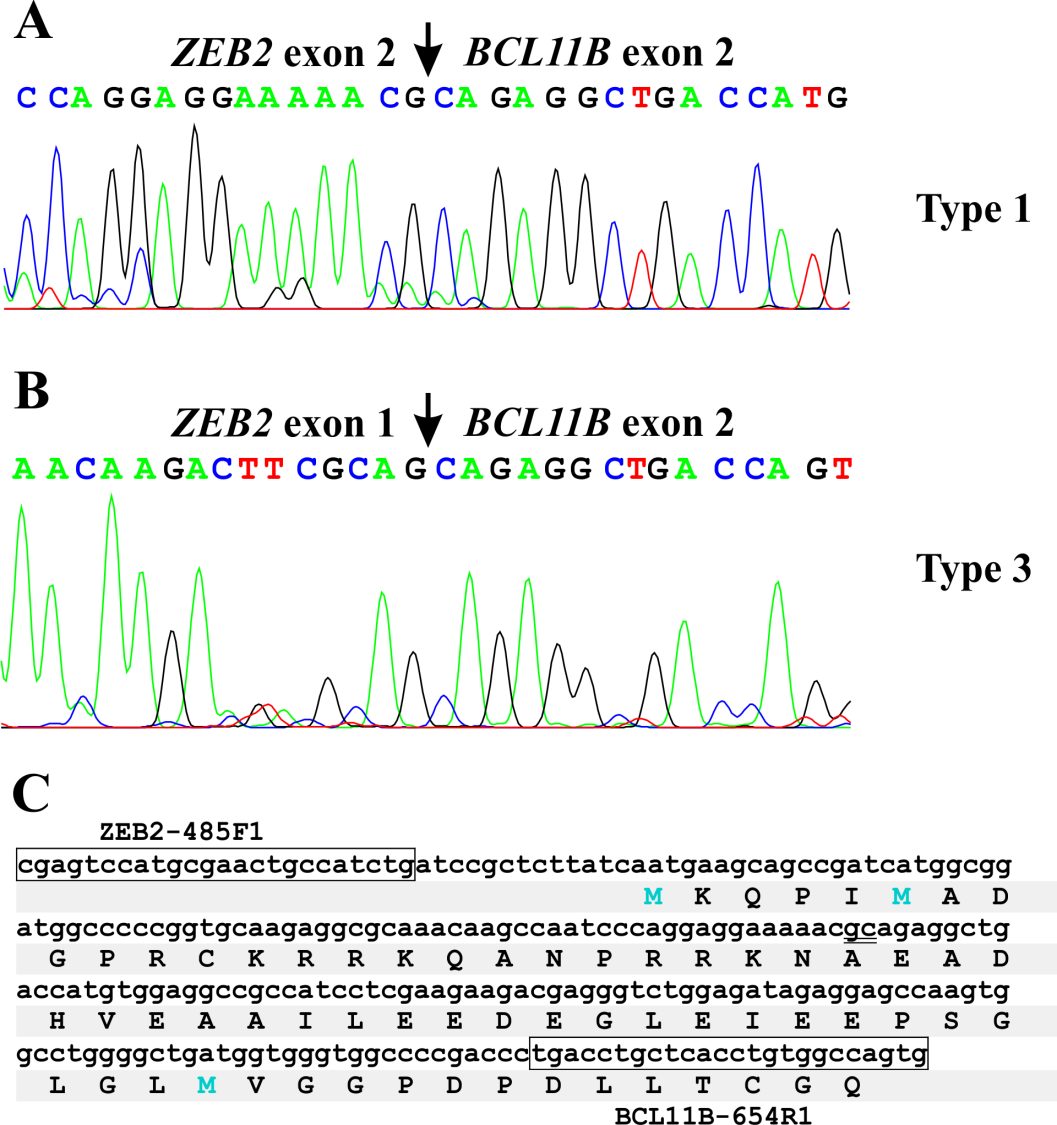
Sanger sequence of the amplified products from the AML patient with the chromosomal aberration t(2;14). A) Partial sequence chromatogram of the cDNA fragment showing that exon 2 of *ZEB2* is fused to exon 2 of *BCL11B* (type 1 fusion transcript). B) Partial sequence chromatogram of the cDNA fragment showing that exon 1 of *ZEB2* is fused to exon 2 of *BCL11B* (type 3 fusion transcript). C) Sequence of the amplified cDNA fragment using the primers ZEB2-485F1 and BCL11B-654R1 (shown in box). The fusion point “gc” is double underlined. The open reading frame is also shown.

PCR with the ZEB2-383F1 and BCL11B-654R1 primer combination amplified two cDNA fragments of size 337 bp and 192 bp only from the cDNA of the patient with t(2;14) (Fig 2). Sanger sequencing of the long, 337 bp, fragment showed that it was a type 1 *ZEB2-BCL11B* chimeric cDNA fragment with the fusion point identical to that found with FusionCatcher, i.e., exon 2 of *ZEB2* (nucleotide 595 in the sequence with accession number NM_014795.3) was fused to exon 2 of *BCL11B* (nucleotide 554 in the sequence with accession number NM_022898.2) (Fig 3A). Sanger sequencing of the short, 192 bp, fragment showed that it was a type 3 *ZEB2-BCL11B* chimeric cDNA fragment in which the first three bases, CAG, of exon 2 of *BCL11B* were also present, i.e., exon 1 of *ZEB2* (nucleotide 453 in the sequence with accession number NM_014795.3) was fused to exon 2 of *BCL11B* (nucleotide 554 in the sequence with accession number NM_022898.2) (Fig 3B).

PCR with the ZEB2-485F1 and BCL11B-654R1 primer combination amplified a 235 bp cDNA fragment only from the cDNA of the patient with t(2;14) (Fig 2). Sanger sequencing showed that it was a type 1 *ZEB2-BCL11B* chimeric cDNA fragment (fusion of exon 2 of *ZEB2* with exon 2 of *BCL11B*) (Fig 3A and 3C). No cDNA amplified product was obtained when *BCL11B* forward and *ZEB2* reverse primers were used, suggesting that *BCL11B-ZEB2* was absent or not expressed (Fig 2).

Normal *ZEB2* cDNA fragments were amplified from the cDNAs of the patient with t(2;14), the AML-control, and the normal bone marrow suggesting that *ZEB2* is expressed in the three samples (Fig 2). *BCL11B* cDNA fragments were amplified from the cDNAs of the AML-control and the normal bone marrow, whereas no *BCL11B* cDNA fragments were amplified from the patient´s cDNA suggesting that *BCL11B* was not expressed or had very low expression in the bone marrow of the patient with t(2;14)-*ZEB2-BCL11B* fusion (Fig 2).

## Discussion

We present a case of AML which had FLT3-internal tandem duplication mutation (FLT3-ITD) and cytogenetic aberrations at diagnosis. The primary cytogenetic abnormality was a t(2;14)(q22;q32) chromosomal translocation which rearranged and fused the *ZEB2* gene in 2q22 with the *BCL11B* gene in 14q32. The translocation resulted in the expression of chimeric *ZEB2-BCL11B* transcripts whereas the reciprocal *BCL11B-ZEB2* fusion was either absent or not expressed.

FLT3-ITD mutation is an important prognostic factor but the prognostic impact must be interpreted against the genetic context [13]. In patients with normal karyotype, FLT3-ITD is associated with poor prognosis [14–17]. In acute promyelocytic leukemia with t(15;17), the prognostic impact of FLT3-ITD mutation is minimal [18]. In other contexts, both the mutant to wild type allelic ratio and the position of the ITD within the *FLT3* gene may carry prognostic impact [19, 20]. The present case of AML turned out to be chemoresistant as it failed three induction courses. The FLT3-ITD mutations might have played a role in this but so may the presence at diagnosis of the chromosomal aberration t(2;14)(q22;q32) and its molecular consequence, the generation of a *ZEB2-BCL11B*. One cannot at present say which is more prognostically important.

The same chromosomal translocation as the one we found, t(2;14)(q22;q32), was recently reported in ETP-ALL [8]. In that study, neither *BCL11B-ZEB2* nor *ZEB2-BCL11B* (nor their fusion transcripts) was detected although the translocation was shown to result in overexpression of *ZEB2* [8].

The *ZEB2* gene (in 2q22) codes for a protein which is a member of the Zfh1 family of 2-handed zinc finger/homeodomain proteins. ZEB2 protein is located in the nucleus and functions as a DNA-binding transcriptional repressor that interacts with activated SMADs, the transducers of TGF-beta signaling, and interacts with the nucleosome remodeling and histone deacetylation (NURD) complex (http://omim.org/entry/605802). ZEB2 and its paralogue ZEB1 play a pivotal role in vertebrate embryogenesis [21]. Recent evidence shows that both proteins also drive the process of epithelial-mesenchymal transition during cancer progression [22]. The two proteins are involved in the control of several cancer cell capabilities, including proliferation, senescence, apoptosis, angiogenesis, resistance to chemotherapy and radiotherapy, and tumor invasiveness and metastasis [22].

The *BCL11B* gene and its paralogue *BCL11A* code for krueppel-like C2H2-type zinc finger proteins of the BCL11 family of transcription factors [23]. Although the specific function of *BCL11B* has not been determined, the encoded protein is known to be a bi-functional transcriptional regulator that acts as a repressor and transactivator [24]. BCL11B protein is essential for T-cell development and for maintenance of T-cell identity; in fact, T-cells acquire NK cell properties upon BCL11B deletion [23]. *BCL11B* gene alterations are related to malignant T-cell transformation in hematological malignancies [24]. The fusion genes *BCL11B-TLX3*, *BCL11B-NKX2-5*, *BCL11B-TRD*, and *IKZF2-BCL11B* have all been reported in T-cell acute lymphoblastic leukemia [24]. An *RN28S1-BCL11B* fusion transcript was also identified in a case of mixed-lineage (T/myeloid) acute leukemia with t(6;14)(q25;q32) [25]. The *RN28S1* gene is not translated to protein and *BCL11B* and *RN28S1* were fused in opposite transcription directions. Thus, disruption of the normal functions of *BCL11B* seemed to contribute to leukemogenesis in this case [25]. *BCL11B* was also involved in a case of AML with a similar chromosomal aberration, a t(6;14)(q25~q26;q32), but there was not enough material to identify the partner genomic locus [26]. Very recently, *BCL11B* was shown to be involved in 14q32 translocations with different chromosomal partners in AML [27].

In the *ZEB2-BCL11B* fusion transcripts 1 and 2, the sequence of *ZEB2* involved codes only for the first 24 amino acids (MKQPIMADGPRCKRRKQANPRRKN) of the ZEB2 protein (starting ATG codon in exon 2, on position 523–525 of the *ZEB2* sequence with accession number NM_014795.3) (Fig 3C). This amino acid sequence does not contain any functional domain except nuclear localization signals (KRRK, PRRK, and PRCKRRK), but replaces the first 19 amino acids of BCL11B (MSRRKQGNPQHLSQRELIT) in the ZEB2-BCL11B chimeric protein. The BCL11B in the ZEB2-BCL11B chimeric protein retains all functional domains, i.e., the 6 krueppel-like Zn-finger domains and the proline-rich region [24]. In the *ZEB2-BCL11B* fusion transcript 3, the untranslated exon 1 of *ZEB2* is fused to exon 2 of *BCL11B*.

Taking all the data into consideration, i.e., presence of *ZEB2-BCL11B* fusion transcript and absence of *BCL11B-ZEB2* fusion transcript and wild type *BCL11B*, we believe that placing all functional domains of *BCL11B* under the control of the *ZEB2* promoter represents the leukemogenic mechanism for the *ZEB2-BCL11B* chimera. Consequently, the principal result should be abnormal expression of *BCL11B*. Integrative genomic analyses of *ZEB2* showed that the gene’s proximal promoter region contains a conserved Ets-Smad-binding CGGAGAC motif as well as bHLH-, POU/OCT-, and HIF1-binding sites [28]. Nevertheless, one cannot rule out the possible importance of the 24 amino acids (MKQPIMADGPRCKRRKQANPRRKN) from ZEB2 in the chimeric protein.

According to the Mitelman Database of Chromosome Aberrations and Gene Fusions in Cancer (http://cgap.nci.nih.gov/Chromosomes/Mitelman, last updated on May 7, 2015), a t(2;14)(q21;q32) chromosome translocation was reported in three cases of AML and a t(2;14)(q23;q32) in one case. No information is at hand about the molecular consequences of the translocation in the four cases, but we surmise that it could be generation of a *ZEB2-BCL11B* fusion gene. The difference between two neighboring bands (2q21 and 2q22 or 2q22 and 2q23) would be close to or beyond the resolution level of G-banding. Worthy of mention is furthermore that all five patients (including the present case) so far reported with the t(2;14) aberration were male; this may or may not be coincidental.

In conclusion, we show here that the chromosomal translocation t(2;14)(q22;q32) in AML results in *ZEB2-BCL11B* fusion transcripts the leukemogenic mechanism of which seems to be that the coding regions of the *BCL11B* gene come under the control of the *ZEB2* promoter.

## Supporting Information

S1 Table Fusion transcripts identified using FusionCatcher. Fusion transcripts detected using FusionCatcher.
(XLSX)

S2 Table Fusion transcripts identified using ChimeraScan. Fusion transcripts detected using ChimeraScan.
(XLSX)
